# Neural regions associated with memories of Recalled Experiences of Death (REDs; authentic Near‑Death Experiences [NDEs]): a preliminary functional MRI study

**DOI:** 10.1016/j.resplu.2026.101332

**Published:** 2026-04-21

**Authors:** José Alonso Ruiz, Mayte Rodríguez-González-Moro, Tomás Segura, Víctor M. Serrano del Pueblo, Salvador D. Aznar-Cervantes

**Affiliations:** a Faculty of Medicine, Catholic University of Murcia (UCAM), 30107 Murcia, Spain; b Faculty of Nursing, Catholic University of Murcia (UCAM), 30107 Murcia, Spain; c Faculty of Medicine, University of Castilla-La Mancha, 02008 Albacete, Spain; d Biotechnology Research Group, Murcian Institute for Agricultural and Environmental Research and Development, 30150 Murcia, Spain

**Keywords:** Functional Magnetic Resonance Imaging (fMRI), Near-Death Experience (NDE), Recalled Experience of Death (RED), Autobiographical memory, Neuroimaging, Blood Oxygen Level Dependent (BOLD)

## Abstract

**Introduction:**

This observational study examined brain activation patterns when people relive their memories of recalled experiences of death (REDs), also known as authentic near-death experience (NDE) memories, using structural and functional MRI under normal cerebral perfusion in participants with clinically documented life-threatening events and loss of consciousness.

**Methods:**

Fifteen participants (7 women, 8 men; aged 18–75) were evaluated using two scanners. A block-design paradigm (recall RED–rest) was applied, with prior psychological training, physiological monitoring, and radiological review. Analysis was performed using SPM25. A whole-brain threshold was applied at an uncorrected level of *p* < 0.001 with a minimum cluster extent of *k* ≥ 10 voxels. Region-of-interest (ROI) analyses were performed using small-volume correction with voxel-wise family-wise error (FWE) correction (pFWE < 0.05).

**Results:**

Significant activations were observed in the precuneus (BA7), posterior cingulate cortex (BA23/31), medial prefrontal cortex (BA10), dorsolateral prefrontal cortex (BA9/46), angular gyrus (BA39), secondary visual cortex (BA18/19), middle temporal gyrus (BA21), and orbitofrontal cortex (BA47). The parahippocampal region (BA36) showed a non-significant trend. No activation was found in primary sensorimotor or limbic areas.

**Discussion:**

The reliving of RED/authentic NDE memories was associated with the engagement of brain networks implicated in autobiographical memory and the default mode network (DMN), suggesting the involvement of introspective and self-referential processes. The observed activation patterns were not consistent with those typically reported for primary sensory hallucinations.

**Conclusions:**

Controlled reliving of memories of REDs/authentic NDEs may represent a useful experimental paradigm for investigating multisensory autobiographical memory and neural processes related to conscious self-experience. These findings are consistent with vivid episodic recollection and differ from neural patterns commonly associated with imagined or false memories.

## Highlights

• Controlled fMRI recall of RED/authentic NDE memories was successfully implemented.
• Consistent activation in precuneus, PCC, MPFC, DLPFC, angular gyrus, secondary visual cortex, MTG, and OFC.
• RED/authentic NDE recall engages autobiographical memory and default mode network regions.
• No activation was observed in primary sensory or limbic cortices.
• Findings support RED/authentic NDE memories as vivid, context‑rich autobiographical recollections.

## Graphical abstract

**Figure:**
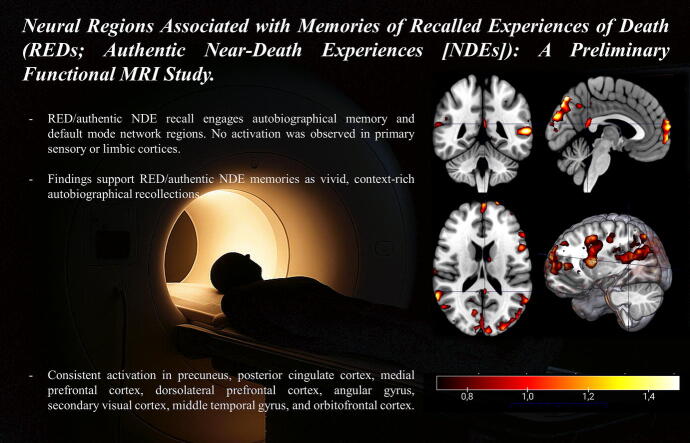

## Introduction

Experiences traditionally referred to as near‑death experiences (NDEs) have been extensively documented for decades in survivors of life‑threatening events. Recent recommendations^1^ proposed the term recalled experiences of death (REDs) to refer specifically to authentic NDEs, defined as specific cognitive and emotional experiences occurring during a period of loss of consciousness (LOC) in relation to a life-threatening clinical event, including but not limited to cardiac arrest.^1^ In this manuscript, “RED” and “authentic NDE” are used interchangeably, as both designate the same phenomenon incorporating the conceptual precision recommended by recent guidelines, while preserving continuity with established NDE literature.

RED/authentic NDE comprise a complex and structured phenomenology including approximately fifty specific experiential elements. These elements are commonly organized into six broad thematic categories: (1) separation from the body accompanied by preserved audiovisual awareness; (2) a transition or movement toward a meaningful destination; (3) a panoramic and morally evaluative life review; (4) a profound sense of ‘homecoming’ or belonging; (5) the return to the physical body; and (6) enduring psychological and behavioral aftereffects.^1^ Importantly, the life review—often central within RED/authentic NDE accounts—extends far beyond ordinary autobiographical recollection: it involves a comprehensive, purposeful, and emotionally salient evaluation of one’s actions, thoughts, and intentions toward others, frequently described as unfolding according to universal moral and ethical standards. Prospective studies conducted in survivors of cardiac arrest and other life‑threatening conditions have documented that some patients report vivid and structured experiences occurring in temporal association with periods of critically reduced or absent cerebral perfusion leading to LOC.^2^, ^3^, ^4^ These observations have contributed to an ongoing scientific debate regarding the mechanisms underlying these phenomena and their relationship to brain function under such ischemic conditions.

Traditionally, RED/authentic NDE have been explored primarily from psychological, phenomenological, and philosophical perspectives. Although advances in neuroscience allow the study of neural activity during the reliving of RED/authentic NDE memories, no neuroimaging modality can capture the neural correlates of the experience as it occurs during cardiac arrest or profound ischemia. Therefore, fMRI studies, including the present work, characterize the neural correlates of memory recall under normal cerebral perfusion rather than the physiological mechanisms during the life‑threatening event itself. A range of explanatory models has been proposed, including neurochemical mechanisms (e.g., endogenous opioid release or ketamine-like dissociation), cortical disinhibition, REM intrusion, and dysfunction of temporoparietal networks.^5^, ^6^ More recently, integrative frameworks such as the NEPTUNE model have been introduced, combining neurobiological and psychological processes to account for the phenomenology of RED/authentic NDE memories.^7^ However, the precise neural mechanisms remain indefinable, partly due to the difficulty of studying these phenomena in real time.

Functional magnetic resonance imaging (fMRI) has emerged as a powerful tool to explore brain activity associated with complex cognitive and introspective states. Unlike structural imaging, fMRI allows the detection of blood-oxygen-level-dependent (BOLD) signal changes, reflecting neural activation patterns with high spatial resolution.^8^ This technique has been successfully applied to study altered states of consciousness, including meditation,^9^ dreaming,^10^ and psychedelic experiences,^11^ among others.

Building on this framework, the present study investigates brain activation patterns during the controlled recall of REDs/authentic NDEs under normal cerebral perfusion, using functional fMRI. The aim is to characterize the neural networks involved in autobiographical memory and self-referential processing associated with memories of REDs/authentic NDEs. Importantly, this approach does not attempt to model neurobiological processes occurring during cardiac arrest or profound hypoxic–ischemic states, but rather to identify the neural substrates engaged during recollection. This distinction represents a major and inherent limitation of the study.

## Methods

### Participants

Fifteen individuals (7 females, 8 males; mean age = 46 years, SD = 17; range: 18–75) were included in the study. All participants had survived a life-threatening clinical event associated with sustained LOC, including documented cardiac arrest and other forms of profound acute medical or neurological compromise resulting in critically reduced or absent cerebral perfusion (e.g., severe traumatic brain injury or acute hypoxic–ischemic states), followed by full neurological recovery. Transient LOC in non–life-threatening contexts (e.g., benign syncope) was an explicit exclusion criterion. Inclusion criteria required the presence of a vivid, consistent experience with phenomenological features compatible with REDs/authentic NDEs.

Prior to imaging, all participants underwent a comprehensive psychological, neurological, and medical evaluation to rule out any functional or clinical conditions that could account for hallucinatory phenomena. Exclusion criteria included a history of psychiatric disorders, consumption of toxic substances, diagnostic uncertainty, paranoid traits (as defined by DSM-5), or severe metabolic or oncological disease. The authenticity and consistency of each participant’s narrative were independently assessed by a team of university-affiliated clinical psychologists using two validated instruments: the most established Greyson NDE Scale^12^ and the Near-Death Experience Content (NDE-C) scale.^13^ Both scales were employed not only to confirm the presence of core experiential features but also to quantify the intensity and phenomenological richness of the reported experiences. In addition, detailed clinical information regarding the index event was obtained from available medical records at two university hospitals and is summarized in Table 1 (see Table S1 for source details).

**Table 1:** Summary of clinical characteristics and core features of REDs/authentic NDEs according to Parnia et al. (2022).^1^

**Patient**	**Age**	**Sex**	**Life-threatening event**	**Cardiac arrest**	**Loss of consciousness**	**Core RED experiential features**
P1	35	F	Cardiac arrest associated with severe cervical trauma	Yes	Prolonged, documented*	OBE; Ineffability; Encounter; Hyperreality
P2	75	M	Cardiac arrest during complicated liver transplantation	Yes	Prolonged, documented*	Perceived death; OBE; Encounter; Hyperreality
P3	24	F	Septic shock secondary to meningococcal meningitis	No	Documented	OBE; Time distortion; Encounter
P4	48	M	Cardiac arrest with severe traumatic brain injury and massive abdominal hemorrhage	Yes	Prolonged, documented*	OBE; Time distortion; Ineffability; Hyperreality
P5	50	F	Cardiac arrest with hypovolemic shock following gastric surgery	Yes	Documented	OBE; Time distortion; Hyperreality
P6	68	F	Cardiac arrest during surgery for peritonitis	Yes	Prolonged, documented*	OBE; Time distortion; Encounter; Hyperreality
P7	40	M	Cardiac arrest following surgery for infectious colitis	Yes	Prolonged, documented*	Time distortion; Encounter; Hyperreality
P8	50	M	Cardiac arrest secondary to intestinal perforation	Yes	Prolonged, documented*	Time distortion; Encounter; Hyperreality
P9	18	M	Severe traumatic brain injury	No	Witnessed	Encounter; Hyperreality
P10	67	M	Severe hypoglycemia following pancreatic surgery for cancer	No	Documented	OBE; Time distortion; Hyperreality
P11	44	F	Cardiac arrest with ischemic event secondary to intestinal bleeding	Yes	Prolonged, documented*	OBE; Time distortion; Ineffability; Life review; Hyperreality
P12	56	F	Cardiac arrest during cardiac surgery	Yes	Prolonged, documented*	OBE; Time distortion; Hyperreality
P13	45	M	Meningitis with loss of consciousness	No	Documented	OBE; Time distortion
P14	24	M	Multitrauma with cardiac arrest	Yes	Prolonged, documented*	OBE; Time distortion; Ineffability; Encounter; Hyperreality
P15	42	F	Intestinal ischemia with cardiac arrest	Yes	Prolonged, documented*	OBE; Time distortion; Hyperreality

Core RED/authentic NDE experiential features include: perceived death, out-of-body experience (OBE), altered perception of time and space, ineffability, encounters with deceased persons or entities, life review or vivid autobiographical recall, and hyperreal clarity.

* Prolonged loss of consciousness indicates sustained unresponsiveness exceeding one hour, as documented in a critical care or resuscitation context.

All participants underwent a structural magnetic resonance imaging (MRI) scan (with contrast administration if structural abnormalities were detected), followed by an fMRI session. During the fMRI acquisition, participants were instructed to remember their RED/authentic NDE. Prior to the fMRI session, participants underwent a structured preparatory training aimed at facilitating vivid autobiographical recall. As part of this training, participants were first instructed to vividly re‑experience a salient, personally meaningful life event unrelated to the RED/authentic NDE. They were guided to recall the event from a first-person perspective, engaging multiple sensory modalities (visual, auditory, olfactory, and, when applicable, somatosensory), and to avoid evaluative, moral, or spiritual interpretations. This procedure was used to familiarize participants with the recall strategy required during scanning.

All procedures were approved by the ethics committees of the participating university and hospitals. Written informed consent was obtained from all participants in accordance with institutional guidelines. Structural and functional MRI data were reviewed by an experienced neuroradiologist using DICOM and MRIcroGL formats.^14^

### Experimental design and procedure

#### Structural MRI acquisition

All participants underwent conventional MRI scanning at two hospitals using Siemens systems, including a Magnetom Vida 3 T (Siemens Healthcare, Germany) and a Magnetom Altea 1.5 T (Siemens Healthcare, Germany). Structural imaging included T1- and T2-weighted sequences in axial and sagittal planes, as well as axial FLAIR sequences. Whole-brain coverage was obtained, with particular attention to potential abnormalities in predefined regions of interest (ROIs). One participant with a detected lesion underwent additional imaging with paramagnetic contrast agent.

#### Functional MRI paradigm

A block design was employed, alternating between activation and rest conditions. During activation blocks, participants were instructed to internally remember their RED/authentic NDE, while rest blocks consisted of passive mental blankness. Prior to scanning, all subjects completed psychological training to ensure accurate and consistent mental imagery during the vivid recollection. Following this preparatory phase, a clinical psychologist conducted an individual assessment to evaluate each participant’s ability to reliably and consistently re-experience autobiographical events using this recall strategy. Only participants who demonstrated stable, coherent, and phenomenologically consistent recall of both the neutral autobiographical event and their RED/authentic NDE memories were included in the fMRI protocol. Physiological parameters (heart rate, blood pressure, and mydriasis) were monitored before and after scanning. Table 2 summarizes the acquisition parameters used during fMRI with each device.

**Table 2:** Acquisition parameters used during fMRI studies by means of both Siemens devices.

	**Magnetom Altea 1.5T**	**Magnetom Vida 3T**
Sequence	Axial EPI	Axial EPI
Slices (No.)/Thickness (mm)	36/3	40/3
Voxel size (mm)	3 × 3 × 3	2.04 × 2.04 × 3
Repetition time/Echo time (ms)	2000/50	2000/30
Flip angle	65°	90°
Field of view (mm)	192	192
Matrix (px)	64 × 64	94 × 94
Bandwidth (Hz/px)	1860	1970
Duration (s)/scans (No.)*	18/9	20/10

* Paradigm: 10 activation blocks alternating with 10 rest blocks, starting with rest. Although block durations varied slightly between scanners, the number of blocks and sequence structure were identical. General Linear Models (GLMs) included only activation blocks, using rest as an implicit baseline.

Although fMRI data were acquired using two different MRI systems (with differing field strengths), all functional images were normalized to a common template and intensity‐standardized to minimize scanner‐specific variability. Following harmonization practices validated in multi‐site fMRI studies,^15^, ^16^ the data were treated as if originating from a single scanner for subsequent group‐level analysis using SPM25 (stand-alone version).

#### Pre-processing of data

Functional data were pre-processed using Statistical Parametric Mapping version 25 (SPM25) including motion correction, slice-timing correction, spatial normalization to MNI space and smoothing with a 6 mm full-width at half-maximum (FWHM) Gaussian kernel, following standard recommendations.^17^, ^18^

#### Statistical analysis

At individual-level (single-subject analysis), functional data were modeled using a block-design general linear model (GLM), contrasting activation blocks (RED/authentic NDE memories recall) against rest (baseline). In this design, the rest condition functions implicitly as the internal baseline control within each subject, allowing the contrast to index task-related activation above that resting baseline. This approach is commonly used in fMRI, where one condition (often rest or fixation) is omitted from the design matrix and thus serves as an implicit baseline.^19^

Each participant’s contrast image (RED/authentic NDE recall > rest) was generated and entered into group-level analyses, in which random-effects analyses were performed using a one-sample *t*-test on individual RED/authentic NDE > rest contrast images (second level), including a binary scanner covariate (1.5 T = 0; 3 T = 1) contrast maps to assess consistent activation patterns across participants.

For multiple comparisons two complementary approaches were implemented, a Whole-Brain Exploratory Analysis and a Small Volume Correction (SVC). The Whole-Brain Exploratory Analysis was conducted to identify unexpected activation patterns. An exploratory threshold of *p* < 0.001 (uncorrected) and a minimum cluster size of *k* ≥ 10 voxels was applied. Peak activations exceeding *T* = 2.65 were reported (Table S3, supplementary material).

Subsequently, priori-defined ROIs were analyzed using SVC based on 4 mm spherical masks centered on coordinates derived from previous literature on resuscitation and NDEs.^20^ Anatomical labels were verified using the BioImage Suite Web MNI ↔ Talairach converter (icbm2tal transformation). The primary ROIs included: precuneus (BA7), posterior cingulate/precuneus (BA23/31), medial prefrontal/frontopolar cortex (BA10), dorsolateral prefrontal cortex (BA9/46), angular gyrus (BA39), secondary visual cortex (BA18/19), middle temporal gyrus (BA21), hippocampal/parahippocampal region (BA36), and the orbitofrontal cortex (BA47). ROI analyses were conducted using small-volume correction with voxel-wise family-wise error (FWE). Both, a significance threshold of pFWE < 0.05, and a minimum cluster size of 6–8 voxels within the ROI mask were considered as statistically significant activations. Mean values were analyzed for different ROIs, based on the BOLD signals across selected Brodmann areas (BAs), and group analyses were conducted (t-tests, ANOVA).

#### Methodological transparency and quality assurance

Methodological reporting adhered to the COBIDAS-fMRI guidelines,^21^ ensuring transparency and reproducibility. Quality control included head-motion evaluation using framewise displacement (FD; threshold = 0.5 mm), with no participants exceeding this threshold or being excluded on this basis. The six realignment parameters were included as nuisance regressors at the first level. Data were acquired on two MRI scanners (Siemens Magnetom Altea 1.5 T; Magnetom Vida 3 T) using comparable EPI protocols and identical block-design paradigms. All functional images were normalized to MNI space and intensity-standardized. A binary scanner covariate (1.5 T = 0; 3 T = 1) was included at the second level to control for site-related variance. A quality control comparison showed no significant differences in age, sex distribution, or mean FD between scanners (all *p* > 0.1). No participants were excluded due to technical, anatomical, or motion-related issues. To assess sensitivity, a post-hoc power analysis (G*Power 3.1) for a one-sample *t*-test (two-tailed, *α* = 0.05, *n* = 15) indicated 55% power to detect medium effects (Cohen’s *d* = 0.5) and 85% power for large effects (*d* = 0.8).

## Results

### Participants

Participants survived life-threatening clinical events associated with documented LOC, including cardiac arrest, severe shock states, traumatic brain injury, central nervous system infections, and metabolic disturbances. While cardiac arrest occurred in most cases, several participants fulfilled RED/authentic NDE criteria without cardiac arrest, consistent with the 2022 consensus framework.^1^ All participants scored above 7/32 on the established Greyson NDE Scale^12^ (Table 3), with a mean score of 17 ± 4, confirming that all cases met the threshold for consensus-defined RED/authentic NDE memories.^1^ Individual item scores for each participant on the Greyson NDE Scale can be consulted in the supplementary material (Table S2). Additionally, results from the validated NDE-C scale revealed scores above 27/80 in all patients, with a mean of 48 ± 10, indicating a consistently high level of phenomenological richness across the sample.^13^

**Table 3:** Greyson near-death experience scale scores.

**Patient**	**Greyson score (0–32)**	**Greyson ≥ 7**
P1	19	Yes
P2	12	Yes
P3	12	Yes
P4	18	Yes
P5	15	Yes
P6	18	Yes
P7	21	Yes
P8	15	Yes
P9	12	Yes
P10	17	Yes
P11	25	Yes
P12	16	Yes
P13	16	Yes
P14	26	Yes
P15	17	Yes

**Summary**. Mean Greyson score: 17.3 ± 4.3. Range: 12–26.

The fifteen participants underwent clinical and neurological follow-up for a period exceeding two years (range: 2–10 years). One patient died one year after the initial evaluation. None of the participants exhibited long-term neurological or psychological sequelae. One individual developed mild-to-moderate alcohol use disorder two years after the study. None of the participants used psychotropic medications or illicit drugs during the follow-up period.

### Structural magnetic resonance imaging

Structural MRI analysis revealed no morphological, volumetric, or hemispheric asymmetries suggestive of disease or genetic abnormalities. No moderate or severe lesions were detected.

**Fig. 1:**
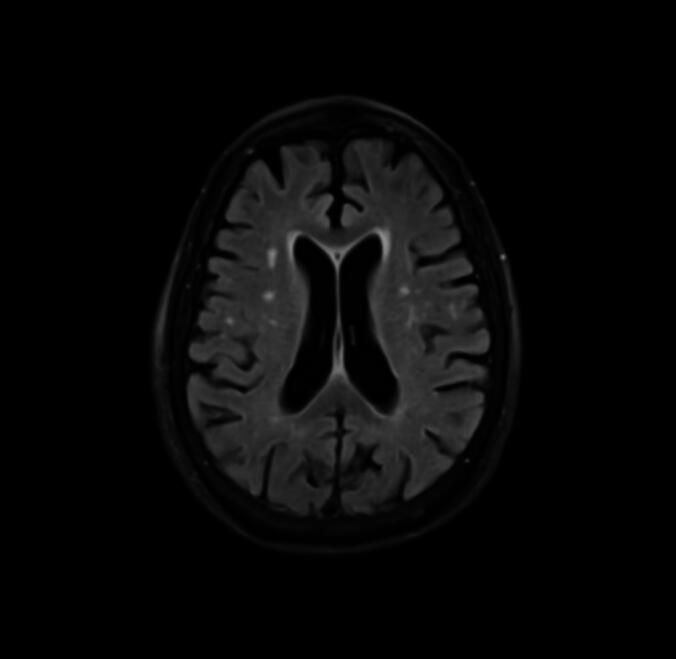
**Structural Magnetic Resonance Imaging (MRI) in FLAIR sequence showing incidental findings of focal hyperintense lesions in the white matter. These alterations are consistent with non-sclerotic inflammatory lesions, without pathology correlation. None of these findings directly affected the predefined regions of interest**.

Incidental findings included: three participants over the age of 60 presented mild lacunar infarcts in the basal ganglia, two of whom also exhibited mild temporal cortical atrophy; one case had a history of diffuse axonal injury with residual microinfarcts in the left basal ganglia; two individuals over 55 years of age showed mild white matter hyperintensities in the centrum semiovale (FLAIR), without radiological criteria for demyelination (Fig. 1); and one subject (a 68-year-old woman) presented a benign cerebellar cavernoma. None of these findings directly affected the predefined regions ROIs.

### Functional magnetic resonance imaging

During RED/authentic NDE recall, the exploratory whole-brain analysis (Table S3) revealed subthreshold trends of activation (*Z* > 2.2, *k* ≥ 10) in multiple regions associated with memory processing, multimodal integration, and the default mode network. Notable among these were the inferior frontal gyrus (BA44/45), dorsolateral prefrontal cortex (BA9/46), medial frontopolar cortex (BA10), posterior cingulate/precuneus (BA23/31), secondary visual cortex (BA18/19), angular gyrus (BA39), precuneus/superior parietal lobule (BA7), middle temporal gyrus (BA21), and hippocampal/parahippocampal regions (BA36).

**Fig. 2:**
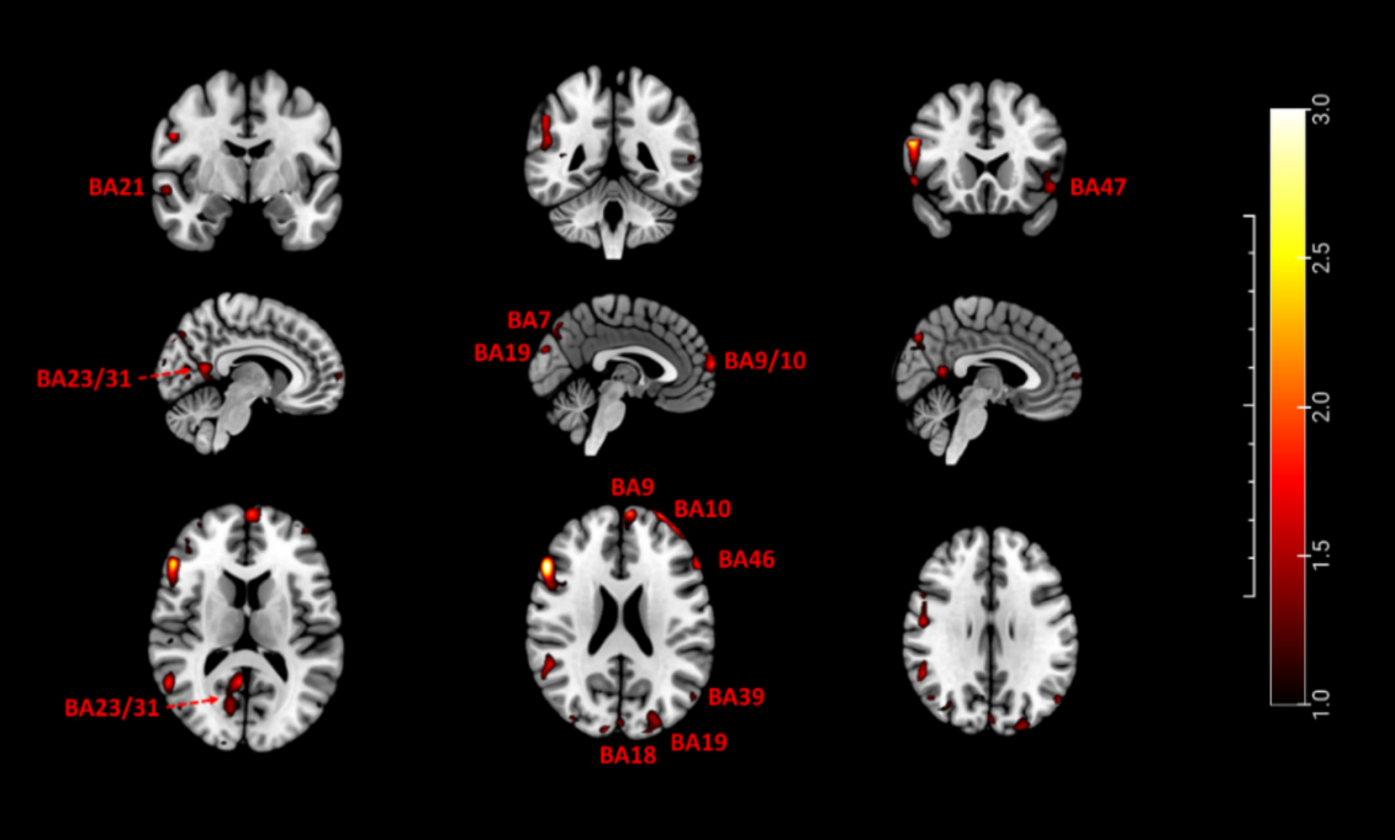
**Brain activation maps during the controlled recall of RED/authentic NDE memories, as measured by functional MRI. The figure displays sagittal, coronal, and axial sections averaged across participants, highlighting the most significantly activated regions (pFWE < 0.05). Note: In the coronal and axial slices, the left side of the image corresponds to the patient's right hemisphere, following radiological convention**. (For interpretation of the references to color in this figure legend, the reader is referred to the web version of this article.)

In contrast, ROI analyses (Table 4) demonstrated robust and consistent activations (all pFWE < 0.05) in the precuneus (BA7), posterior cingulate cortex/precuneus (BA23/31), medial prefrontal/frontopolar cortex (BA10), dorsolateral prefrontal cortex (BA9/46), angular gyrus (BA39), occipital visual cortex (BA18/19), lateral orbitofrontal cortex (BA47), and middle temporal gyrus (BA21). Hippocampal/parahippocampal activation (BA36) showed only a non-significant trend (*p* = 0.06). Patterns were highly consistent across subjects, with no involvement of primary sensorimotor or limbic areas (Fig. 2).

**Table 4:** Brain regions (ROIs) activated during the reliving of RED/authentic NDE memories under SVC analysis (*n* = 15, pFWE < 0.05). “L” and “R” indicate the left and right hemispheres, respectively.

**Brain region (BA)**		**MNI coordinates**			***Z*-value**	**pFWE**
		***X***	***Y***	***Z***		
Precuneus (7)	L	−28	−76	40	3.12	0.008
Posterior cingulate cortex/precuneus (23/31)	R	18	−54	20	2.87	0.015
Occipital visual cortex V2/V3 (18/19)	L	−28	−82	42	2.90	0.014
Angular gyrus, inferior parietal lobule (39)	L	−48	−62	26	2.93	0.013
Medial prefrontal/frontopolar cortex (10)	L	−12	68	8	2.92	0.013
Dorsolateral prefrontal cortex (9/46)	L	−48	28	18	2.82	0.017
Lateral orbitofrontal cortex (47)	L	−38	36	−12	3.11	0.008
Middle temporal cortex (21)	R	64	−6	−4	3.00	0.011
Hippocampus/Parahippocampal cortex (36)	R	10	−6	0	2.24	*0.061

* ROI not activated in a statistically significant way, but considered a trend.

It is noteworthy that the majority of significant activations were localized in regions corresponding to the left cerebral hemisphere, with the exception of Brodmann areas 23/31 and 21, which showed predominant activation in the right hemisphere.

## Discussion

This study suggests that the recall of REDs/authentic NDEs is associated with the engagement of brain networks implicated in episodic autobiographical memory and the DMN, consistent with introspective and self-referential processing. Unlike psychiatric hallucinations or Charles Bonnet syndrome, which typically involve primary sensory cortex, the reliving of RED/authentic NDE memories here were associated with secondary and multimodal regions, with no activation of primary visual or auditory areas. We also observed greater left‑hemisphere involvement, particularly in posterior parieto‑temporal regions linked to multisensory integration and self‑related processing. These aspects are further detailed below for each region showing significant activation during RED/authentic NDE recall.

This study does not aim to model the neurobiology of cardiac arrest or hypoxic–ischemic injury *per se.* Rather, it investigates the neural correlates of reliving REDs/authentic NDEs under conditions of normal cerebral perfusion. This distinction represents a major and inherent limitation of the study. Additionally, the relatively small sample size represents an important limitation, reflecting the challenge of recruiting participants who meet strict criteria for clinically verified REDs/authentic NDEs and are willing to participate in research of this nature, given the social and personal implications associated with such experiences.

Our results partially complement and extend the findings of Beauregard et al. (2009),^20^ by identifying activations in key regions previously described during meditative states evoking religious, spiritual, or mystical experiences associated with REDs/authentic NDEs. However, they differ in two relevant aspects: the predominance of left-hemisphere activations in our cohort, and the absence of activation in the right temporal lobe (BA37), a region that in that study was linked to spirituality-related processes.

The precuneus, primarily composed of **BA7** and part of **BA31**, both activated in the present work, exhibits a functional subdivision between an anterior region involved in self-centered mental imagery strategies, and a posterior region that facilitates successful retrieval of episodic memory.^22^ It is important to highlight that both BA7 and BA31 are part of the Default Mode Network (DMN). The latter has been associated with remote memory retrieval in previous studies.^23^ Recent intracranial stimulation findings similarly indicate that perturbing the anterior precuneus can trigger dissociative states, suggesting that several cortical regions may contribute to these experiences.^24^ However, many factors remain unexplained in this regard, such as the detailed accounts from patients in cardiac arrest who describe events occurring in the room during resuscitation—an aspect that will not be addressed here, as it falls outside the scope of the present investigation.

On the other hand, the posterior cingulate cortex, specifically **BA23**, significantly activated in our study, has been linked in the scientific literature to the re-experiencing of highly vivid memories,^25^ and its crucial role in the integration of emotion and memory during autobiographical recall has also been described.^26^

**BA18** and **BA19**, which showed significant activation in this study, are key for complex visual processing and imagery. fMRI research has confirmed their involvement in the visual simulation of autobiographical scenarios during prospective episodic memory tasks.^27^ Moreover, greater brain BOLD signals has been reported for true memories compared to false ones in the visual occipital regions (BA18 and BA19), as well as in the posterior portion of the hippocampal gyrus (BA36), which also showed a trend-level activation in the present study. In contrast, false memories tend to elicit greater activation in the anterior cingulate gyrus (BA24), where no activation was detected in our study.^28^ Similarly, the activation of BA18 and BA19 has been associated with vividly recollected remote memories, as reported in the work of Gilboa et al. (2004).^25^ These results align with those reported by Palmieri et al. (2014), who showed that EEG activity during the induced recall of RED/authentic NDE memories resembled the neural profile observed during the recollection of true autobiographical memories rather than that associated with false events.^29^

The angular gyrus (**BA39**) showed significant activation in this study. Previous research has found that BA39 and BA40 are more active when recalling true items than false ones, whereas BA38, the cerebellum, and the ventromedial prefrontal cortex tend to be more active during the retrieval of false items. These latter regions did not show activation in the present study.^30^

The medial prefrontal cortex (MPFC) or frontopolar cortex (**BA10**), one of the most evolutionarily advanced brain regions, supports high‑level functions such as metacognition and prospective memory. Its activation in this study is consistent with Beauregard and Paquette (2006), who found MPFC involvement in the metacognitive representation of emotional states during meditative and autobiographical recall tasks.^31^ Similarly, Okado and Stark (2005) linked left BA10 activity to autobiographical memory retrieval.^32^

The left dorsolateral prefrontal cortex (**BA9/BA46**) also showed significant activation. This region is known to support the manipulation of information in working memory tasks across species^33^ and to monitor and maintain temporal order—functions essential for narrating autobiographical events.^34^ The frontal activation observed here is consistent with prior neuroimaging studies linking these areas to higher‑order working memory processes,^30^ response monitoring, and accurate retrieval of source‑related information.

The lateral orbitofrontal cortex (**BA47**), located in the ventrolateral frontal lobe, supports language comprehension, emotional regulation, decision‑making, and socially appropriate behavior. Prior work shows that BA47 helps use distinctive retrieval cues to recover specific long‑term memories.^35^ Thus, remembering remote events such as REDs/authentic NDEs may recruit this region to support strategic memory search^34^, ^36^ and to regulate the emotional responses and behaviors associated with recalling such impactful experiences.

Severe medial temporal‑lobe damage is known to cause profound impairments in forming new declarative memories,^37^ and the role of these structures in memory has been widely demonstrated through fMRI studies.^38^ In our study, the consistent activation of the middle temporal cortex (**BA21**) aligns with previous findings linking this region to episodic and autobiographical memory and to vividly re‑experienced events.^23^, ^25^ Its activation was likewise reported by Beauregard and Paquette (2006) during Carmelite nuns’ recall of their most intense mystical experiences.^31^

Furthermore, the perirhinal cortex (**BA36**), though only showing trend‑level activation, is noteworthy given prior evidence linking it to place and scene processing and to episodic memory, particularly its stronger responses to scenes with rich contextual associations.^39^ Moreover, BA36 activation has been associated with the retrieval of true memories rather than false or illusory ones.^28^, ^30^

Beyond our fMRI findings, other research has examined REDs/authentic NDEs from different angles. Transient bursts of synchronized gamma activity have been observed near death in both animal models^40^ and a human patient,^41^ using EEG, suggesting highly organized brain activity compatible with conscious processing at the edge of death.

Our results show that the recall of RED/authentic NDE memories engages brain regions linked to episodic and autobiographical memory, vivid recollection, visual memory, and the retrieval of true, context‑rich memories. Additional activations involve areas associated with higher‑level cognition, metacognition, and emotional regulation, likely reflecting the impact of these experiences. Altogether, these findings are not consistent with neural activation patterns typically reported during the retrieval of imagined or false events. This interpretation aligns with Thonnard et al. (2013),^42^ as well as Palmieri et al. (2014)^29^ and Moore and Greyson (2017),^43^ all of whom report that RED/authentic NDE memories share the characteristics of genuine autobiographical recall rather than imagined events.

Given the preliminary nature of this study and the limited sample size, further research is necessary to expand upon these findings. Future studies should aim to reproduce the observed activation patterns in larger and more diverse cohorts, integrate longitudinal designs to assess the stability of neural correlates over time, and explore the influence of individual dissimilarities in RED/authentic NDE phenomenology. Moreover, multimodal approaches combining fMRI with EEG or PET could provide complementary insights into the temporal dynamics and neurochemical substrates of RED/authentic NDE recollection.

## Conclusions

This preliminary fMRI study shows that the recall of RED/authentic NDE memories is associated with the engagement of a consistent network of regions involved in episodic and autobiographical memory, multisensory integration, and higher‑order cognition. Key activations were observed in the precuneus, posterior cingulate, medial and dorsolateral prefrontal cortices, angular gyrus, secondary visual areas, and middle temporal cortex, with trend‑level activity in BA36. These activation patterns are consistent with vivid, internally generated autobiographical recollection and differ from neural profiles typically reported for imagined or hallucinatory experiences. While acknowledging the limitations inherent to sample size and the use of memory recall under normal cerebral perfusion, the controlled recall of RED/authentic NDE may represent a valuable paradigm for investigating the neural correlates of autobiographical memory, self-referential processing, and aspects of conscious experience.

## CRediT authorship contribution statement

**José Alonso Ruiz:** Writing – review & editing, Supervision, Methodology, Investigation, Formal analysis, Data curation, Conceptualization. **Mayte Rodríguez-González-Moro:** Writing – review & editing, Investigation, Formal analysis. **Tomás Segura:** Writing – review & editing, Investigation, Formal analysis. **Víctor M. Serrano del Pueblo:** Writing – review & editing, Investigation, Formal analysis. **Salvador D. Aznar-Cervantes:** Writing – original draft, Supervision, Investigation, Formal analysis, Conceptualization.

## Declaration of competing interest

The authors declare no conflict of interest. The present study was carried out independently, during the authors’ personal time, and does not reflect the views or responsibilities of their affiliated institutions.
