# Supplementary material for: Neural regions associated with memories of Recalled Experiences of Death (REDs; authentic Near‑Death Experiences [NDEs]): a preliminary functional MRI study
Source: Resusc Plus. 2026 Apr 21;29:101332. doi: 10.1016/j.resplu.2026.101332 (PMC13147998; doi:10.1016/j.resplu.2026.101332)
Supplement: Supplementary Table S1 [file mmc1.docx]

| **Table S1.** Clinical documentation sources. H1 refers to Hospital Perpetuo Socorro (Cartagena, Spain), and H2 refers to Virgen de la Arrixaca University Clinical Hospital (Murcia, Spain). | | |
| --- | --- | --- |
|  |  |  |
| **Patient** | **Primary hospital** | **Clinical documentation available** |
| P1 | H2 | Full medical record |
| P2 | H2 | Full medical record |
| P3 | H1 | Full medical record |
| P4 | H2 | Full medical record* |
| P5 | H1 | Full medical record |
| P6 | H2 | Full medical record |
| P7 | H2 | Full medical record |
| P8 | H1 | Full medical record* |
| P9 | H1 | Full medical record* |
| P10 | H2 | Full medical record |
| P11 | H2 | Full medical record* |
| P12 | H2 | Full medical record |
| P13 | H2 | Full medical record* |
| P14 | H2 | Full medical record* |
| P15 | H2 | Full medical record |
| *For these patients, clinical documentation included medical records and/or discharge reports originating from external hospitals, which were formally incorporated into the medical records of the participating centers and reviewed by the study investigators. | | |
