# Supplementary material for: Neural regions associated with memories of Recalled Experiences of Death (REDs; authentic Near‑Death Experiences [NDEs]): a preliminary functional MRI study
Source: Resusc Plus. 2026 Apr 21;29:101332. doi: 10.1016/j.resplu.2026.101332 (PMC13147998; doi:10.1016/j.resplu.2026.101332)
Supplement: Supplementary Table S2 [file mmc2.docx]

**Table S2.** Greyson Near-Death Experience Scale – Item-level Scores

| **Patient** | **I1** | **I2** | **I3** | **I4** | **I5** | **I6** | **I7** | **I8** | **I9** | **I10** | **I11** | **I12** | **I13** | **I14** | **I15** | **I16** |
| --- | --- | --- | --- | --- | --- | --- | --- | --- | --- | --- | --- | --- | --- | --- | --- | --- |
| P1 | 2 | 2 | 1 | 2 | 0 | 1 | 0 | 0 | 0 | 2 | 2 | 1 | 1 | 2 | 1 | 2 |
| P2 | 1 | 1 | 1 | 0 | 1 | 0 | 0 | 0 | 1 | 1 | 2 | 2 | 0 | 1 | 2 | 0 |
| P3 | 1 | 1 | 1 | 0 | 0 | 1 | 0 | 0 | 0 | 1 | 2 | 0 | 0 | 2 | 2 | 1 |
| P4 | 2 | 1 | 2 | 2 | 0 | 0 | 0 | 0 | 2 | 1 | 0 | 2 | 2 | 2 | 1 | 1 |
| P5 | 0 | 0 | 2 | 2 | 0 | 1 | 1 | 0 | 0 | 0 | 0 | 0 | 2 | 2 | 2 | 0 |
| P6 | 2 | 1 | 2 | 2 | 0 | 0 | 0 | 0 | 2 | 2 | 0 | 2 | 2 | 1 | 0 | 2 |
| P7 | 1 | 1 | 2 | 0 | 1 | 2 | 0 | 0 | 2 | 2 | 2 | 2 | 2 | 2 | 0 | 0 |
| P8 | 1 | 0 | 2 | 0 | 1 | 1 | 0 | 0 | 2 | 2 | 2 | 0 | 2 | 2 | 0 | 0 |
| P9 | 1 | 1 | 2 | 0 | 0 | 0 | 0 | 0 | 1 | 2 | 2 | 0 | 1 | 2 | 0 | 0 |
| P10 | 2 | 1 | 1 | 2 | 0 | 1 | 0 | 0 | 2 | 2 | 0 | 2 | 2 | 2 | 0 | 0 |
| P11 | 2 | 2 | 2 | 2 | 0 | 1 | 0 | 2 | 2 | 1 | 2 | 2 | 2 | 1 | 0 | 2 |
| P12 | 2 | 1 | 2 | 2 | 0 | 0 | 0 | 0 | 0 | 2 | 1 | 0 | 0 | 2 | 2 | 2 |
| P13 | 0 | 0 | 1 | 0 | 0 | 1 | 2 | 0 | 1 | 1 | 2 | 2 | 2 | 2 | 2 | 0 |
| P14 | 2 | 2 | 2 | 2 | 2 | 2 | 0 | 0 | 2 | 2 | 0 | 2 | 1 | 1 | 2 | 2 |
| P15 | 0 | 0 | 1 | 2 | 1 | 2 | 0 | 0 | 1 | 1 | 0 | 2 | 2 | 2 | 2 | 1 |
