# Supplementary material for: Neural regions associated with memories of Recalled Experiences of Death (REDs; authentic Near‑Death Experiences [NDEs]): a preliminary functional MRI study
Source: Resusc Plus. 2026 Apr 21;29:101332. doi: 10.1016/j.resplu.2026.101332 (PMC13147998; doi:10.1016/j.resplu.2026.101332)
Supplement: Supplementary Table S3 [file mmc3.docx]

**Table S3.** Whole-Brain Exploratory Analysis results. (n=15, p < 0.001). “L” and “R” indicate the left and right hemispheres, respectively.

| **MNI coordinates**  **(X, Y, Z)** | **Hemisphere** | **Brain region** | **BA** | **Z-value** | **p_uncorr** | **kₑ**  **(voxels)** |
| --- | --- | --- | --- | --- | --- | --- |
| (-50, 8, 20) | L | Inferior frontal gyrus, pars opercularis | 44 | 3.22 | 0.001 | 41 |
| (-10, -40, 26) | L/med. | Posterior cingulate cortex/precuneus | 31/23 | 3.12 | 0.001 | 92 |
| (-14, -44, 32) | L./med. | Dorsal precuneus/posterior cingulate cortex | 31 | 3.0 | 0.003 | 15 |
| (-28, 28, 10) | L | Dorsolateral prefrontal cortex (middle frontal gyrus) | 9/46 | 3.0 | 0.001 | 15 |
| (64, -6, -4) | R | Middle temporal cortex | 21 | 3.0 | 0.001 | 6 |
| (-48, -62, 26) | L | Angular gyrus, inferior parietal lobule | 39 (±40) | 2.93 | 0.002 | 12 |
| (-12, 68, 8) | L/med. | Medial prefrontal/frontopolar cortex | 10 | 2.92 | 0.002 | 32 |
| (-28, -82, 42) | L | Dorsal parieto-occipital cortex | 7 (±19) | 2.9 | 0.002 | 14 |
| (-32, -74, 36) | L | Superior parietal lobule / dorsal precuneus | 7 | 2.87 | 0.002 | 15 |
| (-32, -70, 28) | L | Precuneus / superior parietal lobule (SPL), PCC transition zone | 7 (±31) | 2.82 | 0.002 | 91 |
| (-48, 28, 18) | L | Dorsolateral prefrontal cortex (middle frontal gyrus) | 9/46 | 2.82 | 0.004 | 29 |
| (18, -54, 20) | R | Posterior cingulate cortex/precuneus | 31 | 2.79 | 0.007 | 13 |
| (-48, 20, 6) | L | Inferior frontal gyrus, pars triangularis | 45 | 2.78 | 0.003 | 10 |
| (-6, -78, 44) | Med./L | Superior parietal lobule / dorsal precuneus | 7 | 2.78 | 0.004 | 13 |
| (30, -74, 38) | R | Superior parietal lobule / dorsal parieto-occipital cortex | 7 | 2.76 | 0.004 | 13 |
| (-52, 22, 24) | L | Inferior frontal gyrus – middle frontal gyrus (superior opercular/triangular portion) | 44/45 | 2.76 | 0.004 | 10 |
| (14, -62, 26) | R | Posterior cingulate cortex/precuneus | 31 | 2.47 | 0.007 | 12 |
| (-24, -88, 26) | L | Superior occipital cortex / cuneus | 19 | 2.44 | 0.007 | 10 |
| (10, -6, 0) | R | Hippocampus / Parahippocampal gyrus | 36 | 2.24 | 0.013 | 12 |
